# The Elastin Receptor Complex: A Unique Matricellular Receptor with High Anti-tumoral Potential

**DOI:** 10.3389/fphar.2016.00032

**Published:** 2016-03-04

**Authors:** Amandine Scandolera, Ludivine Odoul, Stéphanie Salesse, Alexandre Guillot, Sébastien Blaise, Charlotte Kawecki, Pascal Maurice, Hassan El Btaouri, Béatrice Romier-Crouzet, Laurent Martiny, Laurent Debelle, Laurent Duca

**Affiliations:** UMR CNRS/URCA 7369, SFR CAP Santé, Université de Reims Champagne Ardenne, Faculté des SciencesReims, France

**Keywords:** extracellular matrix, elastokines, ERC, neuraminidase-1, therapeutic targets

## Abstract

Elastin, one of the longest-lived proteins, confers elasticity to tissues with high mechanical constraints. During aging or pathophysiological conditions such as cancer progression, this insoluble polymer of tropoelastin undergoes an important degradation leading to the release of bioactive elastin-derived peptides (EDPs), named elastokines. EDP exhibit several biological functions able to drive tumor development by regulating cell proliferation, invasion, survival, angiogenesis, and matrix metalloproteinase expression in various tumor and stromal cells. Although, several receptors have been suggested to bind elastokines (α_v_β_3_ and α_v_β_5_ integrins, galectin-3), their main receptor remains the elastin receptor complex (ERC). This heterotrimer comprises a peripheral subunit, named elastin binding protein (EBP), associated to the protective protein/cathepsin A (PPCA). The latter is bound to a membrane-associated protein called Neuraminidase-1 (Neu-1). The pro-tumoral effects of elastokines have been linked to their binding onto EBP. Additionally, Neu-1 sialidase activity is essential for their signal transduction. Consistently, EDP-EBP interaction and Neu-1 activity emerge as original anti-tumoral targets. Interestingly, besides its direct involvement in cancer progression, the ERC also regulates diabetes outcome and thrombosis, an important risk factor for cancer development and a vascular process highly increased in patients suffering from cancer. In this review, we will describe ERC and elastokines involvement in cancer development suggesting that this unique receptor would be a promising therapeutic target. We will also discuss the pharmacological concepts aiming at blocking its pro-tumoral activities. Finally, its emerging role in cancer-associated complications and pathologies such as diabetes and thrombotic events will be also considered.

## Cancer Development and Extracellular Matrix

Despite a great progress concerning predictive biomarkers, diagnostic and prognostic strategies, cancer remains the second leading cause of death worldwide after cardiovascular diseases. In 2012, approximately 14 million of new cases and 8.2 million of cancer related deaths have been reported, according to the World Health Organization.

Although, the development of cancer was initially thought to be initiated when a single mutated cell begins to proliferate abnormally leading to the formation of primary tumor (*in situ*), the polyclonal origin of tumors has now been proposed (Parsons, 2008). Malignant cells then cross the tissue, possibly the basement membrane, and invade the extracellular matrix (ECM). From there, invasive tumor cells can spread throughout the body *via* the lymphatic or circulatory systems creating metastatic tumors.

Extracellular matrix remodeling is crucial for regulating tissue homeostasis but also contributes to disease when it is dysregulated. It is composed of macromolecules such as collagens, elastin, laminins, fibronectin, and proteoglycans. Those components interact with cell receptors, transmitting signals that orientate cell adhesion, migration, proliferation, apoptosis, survival, or differentiation. ECM does not only behave as a simple physical support for tissue integrity and plasticity. It is also a reservoir of growth factors, proteases, and other signaling molecules (Hynes, 2009).

During tumor progression, ECM is modified by proteases secreted by both normal and tumor cells. This degradation generates bioactive fragments called matrikines or matricryptines (Davis et al., 2000; Maquart et al., 2004). Matrikines can modulate cell proliferation, migration, invasion, apoptosis, angiogenesis as well as the production and activation of matrix metalloproteinases (MMPs) and the plasminogen system (Bellon et al., 2004; Maquart et al., 2005). In this review, we will focus on elastin, and especially on pro-tumoral activities of elastin-derived peptide (EDP) through their unique receptor, the elastin receptor complex (ERC).

## Elastin

### Elastic Fibers Components

The elastin synthesis, begins during the fetal period (Uitto et al., 1991) and peaks just before birth. Elastogenesis then decreases rapidly to disappear at puberty (Swee et al., 1995). The half-life of elastin is about 70 years (Petersen et al., 2002) and neo-synthesis is low or inexistent. In addition, the ability to form functional elastic fibers is lost. Elastic fibers are essential components of the ECM and are responsible for elasticity of vertebrate tissues. They are found in abundance in tissues subjected to high mechanical stresses requiring repeated cycles of expansion and back to their original state such as the skin, lungs, tendons, or arteries.

Elastic fibers are complex macromolecular assemblies consisting of a coat of fibrillin-rich microfibrils surrounding a heart of elastin (Kielty et al., 2002). The architecture of mature elastic fibers is extremely complex and tissue-specific, reflecting the particular functions they have in tissues. Elastin is a highly hydrophobic polymer of crosslinked-tropoelastin monomers. Microfibrils are made by glycoproteins such as fibrillin-1, fibrillin-2, microfibril-associated glycoprotein-1 (MAGP-1), emilins, latent transforming growth factor β-binding proteins (LTBPs), microfibrillar-associated proteins (MFAPs), and Fibulins (Mithieux and Weiss, 1995). The tropoelastin sequence is composed of alternating domains of very hydrophobic repeating units (which ensure elasticity) and lysine-rich domains. These lysine residues are essential, since the oxidative deamination of their side chains allows the formation of mature elastin covalent crosslinks, i.e., desmosine and isodesmosine, that confer a great mechanical resistance to the elastomer.

### Biosynthesis

Elastin is synthesized and secreted from various cell types such as endothelial cells and fibroblasts (Rodgers and Weiss, 2005).

After a major splicing, mature tropoelastin mRNA is exported out of the nucleus and its translation occurs on the surface of the rough endoplasmic reticulum (RER) forming a polypeptide of about 70 kDa with a N-terminal signal sequence of 26 amino acids which is cleaved when the protein reached the RER lumen (Grosso and Mecham, 1988). After release of the signal peptide, the protein is associated with elastin-binding protein (EBP) to prevent its aggregation and premature degradation (Hinek et al., 1995). The EBP-tropoelastin assembly is then directed to the plasma membrane. EBP is secreted and binding of galactose sugars on its galactolectin site causes the release of tropoelastin, which is then aligned and properly incorporated into the growing elastic fiber (Privitera et al., 1998). After tropoelastin release, EBP is recycled and can accompany another tropoelastin molecule.

## Elastin Degradation and Elastin Peptides

Elastases cleave insoluble and soluble elastin and include serine-, cysteine-, and metallo-proteinases. The serine proteinases neutrophil elastase (Ela-2), cathepsin G, and proteinase-3 and four members of the cysteine cathepsin family (L, S, K, and V) display elastinolytic activity. Moreover, four MMP are elastases (MMP-2, MMP-7, MMP-9, MMP-12). Some generated EDP harbor a GxxPG consensus motif (where x represents any amino acid) adopting a type VIII β-turn, essential for their bioactivity (Brassart et al., 2001). These bioactive EDP are referred as elastokines and the typical elastokine is the VGVAPG peptide, found in the domain encoded by exon 24 of human tropoelastin. Other bioactive GxxPG motifs, GVYPG, GFGPG and GVLPG, and longer elastokines have been reported (Heinz et al., 2012). For instance, MMP-7, -9, and -12 have been shown to generate the bioactive peptides YTTGKLPYGYGPGG, YGARPGVGVGGIP, and PGFGAVPGA (Heinz et al., 2010).

Elastokines contribute to cancer progression by stimulating several capacities of tumor cells such as an elevated expression and secretion of proteases, strongly potentiating their migration and matrix invasion properties (Brassart et al., 1998; Ntayi et al., 2004; Coquerel et al., 2009; Toupance et al., 2012; Donet et al., 2014; **Table 1**). Interestingly, elastokines present potent chemotactic activity on melanoma cells and their presence at a distant organ might contribute to metastasis (Pocza et al., 2008). EDP have also been reported to induce *in vitro* proliferation of glioblastoma (Hinek et al., 1999), and astrocytoma human cell lines (Jung et al., 1998) as well as murine melanoma cell line (Devy et al., 2010). Our laboratory was the first to demonstrate *in vivo* that EDP enhanced murine melanoma cells growth and invasion (Devy et al., 2010).

**Table 1:** Cancer-associated biological effects of EDP.

Biological effects	Cell types	EDPs cancer-associated biological effects
Angiogenesis	Endothelial cells	Nackman et al., 1997; Robinet et al., 2005; Daamen et al., 2008; Fahem et al., 2008; Gunda et al., 2013
Apoptosis and cell survival	Fibroblasts	Cantarelli et al., 2009
	Lymphocytes	Péterszegi and Robert, 1998; Péterszegi et al., 1999
Adhesion	Fibroblasts	Hornebeck et al., 1986; Groult et al., 1991; Yamamoto et al., 2002; Rodgers and Weiss, 2004; Bax et al., 2009; Akhtar et al., 2011
	Astrocytoma	Jung et al., 1999
	Carcinoma	Timar et al., 1991; Svitkina and Parsons, 1993
	Melanoma	Timar et al., 1991; Svitkina and Parsons, 1993
Proliferation	Fibroblasts	Ghuysen-Itard et al., 1992; Kamoun et al., 1995; Tyagi et al., 1996; Tajima et al., 1997; Duca et al., 2005; Shiratsuchi et al., 2010
	Lymphocytes	Poggi and Mingari, 1995; Péterszegi et al., 1996
	Melanoma	Devy et al., 2010
	Astrocytoma	Jung et al., 1998
	Glioma	Hinek et al., 1999
	Endothelial cells	Ito et al., 1998; Dutoya et al., 2000
Tumor invasion and proteases release	Fibroblasts	Gminski et al., 1991a,b; Archilla-Marcos and Robert, 1993; Landeau et al., 1994; Brassart et al., 2001; Huet et al., 2001
	Endothelial cells	Robinet et al., 2005; Fahem et al., 2008; Siemianowicz et al., 2010, 2015
	Monocytes	Fülöp et al., 1986; Varga et al., 1997
	Lymphocytes	Péterszegi et al., 1996, 1999
	Melanoma	Ntayi et al., 2004; Pocza et al., 2008; Devy et al., 2010
	Glioma	Coquerel et al., 2009
	3LL-HM carcinoma	Timar et al., 1991
	Lung cancer	Toupance et al., 2012
	HT1080 fibrosarcoma	Brassart et al., 1998; Huet et al., 2002;Donet et al., 2014
Chomotaxis and migration	Keratinocytes	Fujimoto et al., 2000
	Fibroblasts	Senior et al., 1982, 1984; Mecham et al., 1989; Grosso and Scott, 1993b; Duca et al., 2005; Shiratsuchi et al., 2010
	Eodothelial cells	Long et al., 1989; Skeie and Mullins, 2008; Skeie et al., 2012
	Monocytes	Senior et al., 1980, 1984; Bisaccia et al., 1994; Castiglione Morelli et al., 1997; Uemura and Okamoto, 1997; Hance et al., 2002; Houghton et al., 2006
	Macrophages	Kamisato et al., 1997; Guo et al., 2006, 2011
	3LL-HM carcinoma	Timar et al., 1991
	M27 lung cancer	Blood et al., 1988; Blood and Zetter, 1989, 1993; Yusa et al., 1989; Grosso and Scott, 1993a
	Melanoma	Mecham et al., 1989; Pocza et al., 2008
	HT1080 fibrosarcoma	Donet et al., 2014

Elastokines have also biological effects on normal cells. They stimulate migration and proliferation of monocytes and skin fibroblasts (Senior et al., 1984; Shiratsuchi et al., 2010). They up-regulate MMP expression by fibroblasts inducing a remodeling program in favor of melanoma cell invasion. Elastokines exhibit pro-angiogenic activity through MT1-MMP and NO-mediated increase of endothelial cell migration and tubulogenesis (Robinet et al., 2005; Fahem et al., 2008; Gunda et al., 2013). A wide range of biological effects on immune cells was reported in response to EDP stimulation (Antonicelli et al., 2007). Among them, the chemotactic activity (Nowak et al., 1989; Hance et al., 2002; Houghton et al., 2006; Guo et al., 2011) and elastases production (Hauck et al., 1995; Péterszegi et al., 1997; Varga et al., 1997) amplify elastolysis and increase inflammatory cells recruitment at the tumor site.

## Receptors and Signaling

The biological effects of EDP are mediated by their binding to their cell surface receptors. Among them, the ERC is the most prominent but others potential receptors have also been reported, namely galectin-3 (Pocza et al., 2008) and integrins α_v_β_3_ and α_v_β_5_ (Rodgers and Weiss, 2004; Lee et al., 2014b).

Galectin-3 is expressed in normal and tumor cells and possesses diverse biological functions associated with inflammatory response such as adhesion, cell differentiation, cell migration, and cytokine production. It also modulates others biological functions linked to tumor development like angiogenesis, tumor progression, proliferation, chemotactic, and cell-matrix interactions (Fortuna-Costa et al., 2014). It was moreover reported that galectin-3 is able to interact with both soluble and insoluble elastin in a lactose-dependent manner (Ochieng et al., 2004). This interaction can modulate tumor development as observed by the ability of some specific EDP, such as VGVAPG and VAPG, to amplify melanoma invasion (Pocza et al., 2008).

Integrin α_v_β_3_ regulates diverse biological functions such as cell adhesion, proliferation and migration (Byzova et al., 1998). α_v_β_3_ mainly binds ligands through RGD sequence recognition but it can also recognize others ligands that do not harbor this motif. Indeed, integrin α_v_β_3_ recognizes with high affinity the RKRK sequence present in the C-terminal domain of tropoelastin (Bax et al., 2009). Moreover, a recent study has shown the ability for α_v_β_5_ to bind tropelastin involving the central region of the protein (Lee et al., 2014b).

**FIGURE 1:**
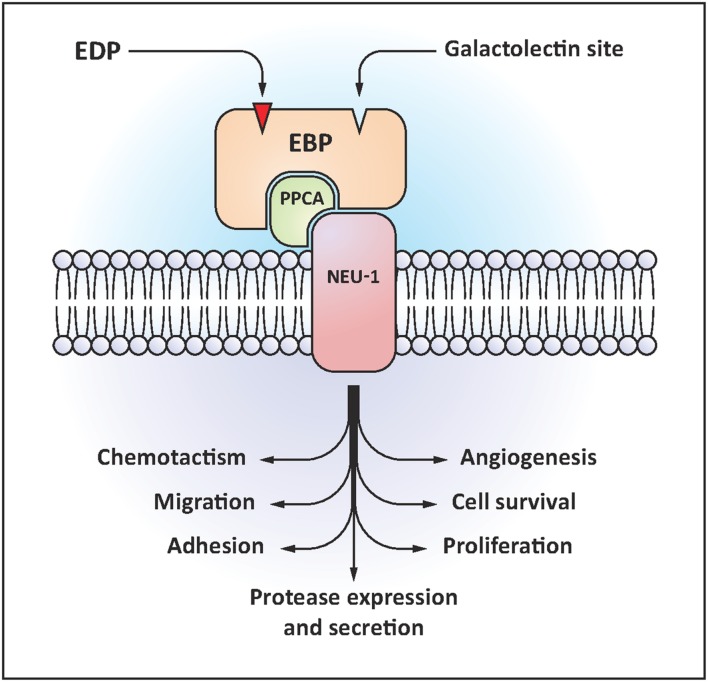
**Elastin receptor complex (ERC) structural organization and EDP-induced biological effects**.

The ERC is a heterotrimeric receptor binding elastokines presenting the GxxPG consensus sequence (**Figure 1**). This receptor contains a peripheral 67-kDa protein named EBP (accession number P16278-2), and two membrane-associated proteins, the protective protein/cathepsin A (PPCA, 55-kDa, accession number P10619) and neuraminidase-1 (Neu-1, 61-kDa, accession number Q99519; Duca et al., 2007). EBP is an enzymatically inactive spliced variant of lysosomal β-galactosidase (Privitera et al., 1998). EBP possesses two functional binding sites: the elastin site on which EDP binding triggers signaling pathways, and the galactolectin site whom occupancy by galactosugars induces EDP release and dissociation of the complex (Mecham et al., 1991). When EDP bind to EBP, neuraminidase-1 is activated and catalyzes the desialylation of adjacent gangliosides such as GM_3_ [*N*-acetylneuraminic-α-(2-3)-galactosyl-β-(1-4)-glucosyl-(1-1′)-ceramide] generating lactosylceramide (LacCer) production (Rusciani et al., 2010; Scandolera et al., 2015). LacCer is a second messenger able to activate intracellular signals. Intracellular signaling pathways modulated by EDP depend on the cell type. Duca et al. (2002) showed that pro-MMP-1 induction mediated by EDP in human dermal fibroblasts involves the activation of MEK1/2/ERK1/2 pathway through a signal dependent on PKA and PI3K. Moreover, complementary works demonstrated that EDP are able to modulate signaling pathways involving modules such as Ras-Raf-1-MEK1/2-ERK1/2, Gi-p110γ-Raf-1-MEK1/2-ERK1/2, cAMP-PKA-B-Raf-MEK1/2-ERK1/2, NO-cGMP-PKG-Raf-1-MEK1/2-ERK1/2 or Gi-p110γ-Akt-caspase9-Bad-Foxo3A. They also induce Ca^2+^ mobilization (Jacob et al., 1987; Faury et al., 1998; Duca et al., 2005; Fahem et al., 2008).

Although EDP are the main ligands of ERC, bioactive xGxxPG motifs are found in numerous matrix protein sequences. For instance, laminin B1 chain harbors a LGTIPG sequence that triggers elastin-like signaling, inducing pro-tumoral activities, and was identified as a ligand of this receptor in melanoma cells. That is why EBP was first called the 67-kD elastin/laminin binding protein (Mecham et al., 1989; Hinek, 1994).

## Anti-ERC Therapeutic Strategies

Limiting or blocking the deleterious effects of EDP/ERC interaction can be achieved either by limiting EDP generation or by acting directly on the ERC and its signaling pathways. As this review is focused on ERC, we will not detail here elastases inhibition strategies.

### Targeting EBP

Blocking the binding of EDP on EBP can be achieved either by using the V14 peptide or a galactoside. The V14 peptide (VVGSPSAQDEASPL) is derived from EBP sequence and can bind EDP. As a consequence, excess V14 can trap circulating EDP thereby blocking their effects (Robinet et al., 2007). Alternatively, the use of galactosugars (mostly lactose or chondroitin sulfate) leads to the shedding of EBP from the complex and blocks the corresponding signaling (Blaise et al., 2013). Although V14 and galactosugars helped to better understand EDP biology, their selective delivery at the site of vascular injury is still an issue.

### Targeting Neu-1

The catalytic activity of Neu-1 is required for proper EDP signaling. As a consequence, its inhibition blocks EDP-driven signals. The 2-deoxy-2,3-dehydro-*N*-acetylneuraminic acid (ddNeu5Ac) inhibitor is currently used as a sialidase inhibitor to block EDP effects (Duca et al., 2007). However, this compound also inhibits other sialidases precluding its therapeutic use.

An attempt was made to design and synthesize inhibitors for human neuraminidases (Magesh et al., 2009) but the results were not satisfactory. Indeed, selectivity was not achieved, probably because the structures of human sialidases are not fully described.

Recently, O’Shea et al. (2014) used oseltamivir phosphate to target Neu-1 and disable cancer cell survival in human pancreatic cancer with acquired chemoresistance. This study suggests that Tamiflu could be possibly used to selectively block Neu-1.

### Blocking EDP-Mediated Signaling Pathways

In human skin fibroblasts, Neu-1 promotes the local conversion of the GM_3_ ganglioside into LacCer following EDP treatment. LacCer can therefore be regarded as the second messenger of the complex (Rusciani et al., 2010). Thus, blocking the signaling pathways triggered by LacCer will suppress EDP effects. In this context, PI3Kg is a promising target as this kinase is central to EDP-related signaling (Duca et al., 2005).

Besides this direct signaling, Neu-1 is also known for its ability to desialylate other membrane residing glycoconjugates, notably receptors. During the last decade, Neu-1 has been shown to modulate insulin receptor signaling (Blaise et al., 2013) and to regulate TLR4 (Amith et al., 2010), Trk A (Jayanth et al., 2010), PDGF-BB and IGF receptors (Hinek et al., 2008), EGF and MUC1 receptors (Lillehoj et al., 2012), and CD31 (Lee et al., 2014a). Consequently, this ERC subunit now emerges not only as a catabolic enzyme but also as a regulator of signaling platforms (Pshezhetsky and Hinek, 2011).

Efforts are now made to understand the intricate network of Neu-1 partners and how they interact each other in order to devise new strategies aiming at selectively impeding these interactions.

## ERC Involvement in Cancer-Associated Processes

### Diabetes

Type 2 diabetes leads to many micro- and macrovascular complications implicating several molecular factors and with significant impact in terms of morbidity and mortality. For example, type 2 diabetes mellitus is associated with an increase in the expression of MMPs, especially MMP-2 and 9, and an increase in the degradation of elastin and, thus, the generation of EDP (Hopps and Caimi, 2012). EDP immunogenic properties favor the formation of anti-elastin antibodies, which concentrations are greatly increased in diabetic patients as compared to non-diabetic subjects (Fulop et al., 1990).

Cancer is a well-known complication of diabetes. Indeed, cancer development is more frequent in diabetic people than in the general population. According to recent studies and meta-analyzes, cancers involving the pancreas (Morrison, 2012), liver (Giovannucci et al., 2010), colon (Larsson et al., 2005), breast (Larsson et al., 2007), urinary tract (Larsson et al., 2006), and the endometrium (Friberg et al., 2007) occur more frequently among patients with type 2 diabetes. In contrast, a recent meta-analysis (Giovannucci et al., 2010) involving a total of nineteen studies, indicates a reduced risk of occurrence of 16% for prostate carcinoma in diabetic patients.

Several mechanisms could be involved in the initiation and/or progression of cancer in diabetes but these mechanisms still remain hypothetical.

Insulin and its associated receptor seem to have a key role, as well as the insulin-like growth factor 1 and its receptor, in the interplay between cancer and diabetes (Cohen and LeRoith, 2012). Furthermore, hyperglycemia could promote tumor progression due to increased intracellular metabolic activity specific to cancer cells and a greater membrane transport of glucose. Interestingly, it has been shown that the activation of pro-tumoral factors such as neutrophil elastase (NE; Moroy et al., 2012) and the accumulation of EDP in blood may represent inducible factors of insulin resistance in mice (Blaise et al., 2013). Indeed, NE^-/-^ mice have increased blood glucose, decreased insulin pathway activity, and increased gluconeogenesis (Talukdar et al., 2012). This insulin resistance might be due to a decrease in the expression of Hsp90 and an increase of the inhibitory protein (IkB) of the transcription factor NFkB. The pro-inflammatory state present in diabetics could decrease the efficiency of intracellular antioxidants and also participate in carcinogenesis. Some cytokines, such as tumor necrosis factor α (TNF-α), promote tumor growth by activating NF-κB (Szlosarek et al., 2006). Another mechanism related to the pro-inflammatory state, mitochondrial dysfunction, would be present in diabetic patients resulting in decreased energy available for DNA repair. Meanwhile, our laboratory has shown that EDP, which are products of NE activity, induce hyperglycemia and insulin resistance in animals by inhibiting insulin receptor signaling pathways in muscle, liver, and adipose tissue. Although the precise mechanism remains to be elucidated, it appears that this inhibitory effect involves a physical interaction between the insulin receptor and the ERC *via* its Neu-1 subunit (Blaise et al., 2013). Consequently, the ERC could not only exhibit a clear pro-tumoral aspect, but is also involved in the outcome of diabetes influencing cancer development.

### Thrombosis

Cancer-associated thrombosis is a major cause of morbidity and mortality in patients with cancer. Thrombotic complications, mostly from venous thromboembolism, are the second cause of death among patients with cancer (Khorana et al., 2007). Several mechanisms have been suggested to contribute to these increased thrombotic complications such as the prothrombotic activity of cancer cells (Mitrugno et al., 2015), the secondary deleterious effects of anti-cancer therapies and the interaction of cancer cells with blood platelets. Indeed, cumulative evidences show that platelets and their activation play important roles in cancer growth and dissemination (Gay and Felding-Habermann, 2011). Therefore, antiplatelet therapy to minimize platelet activation and aggregation, typically reserved for cardiovascular diseases, may have profound implications in cancer treatment (Franco et al., 2015).

In a recent study published by Kawecki et al. (2014), EDP were shown to decrease human platelet aggregation in whole blood and washed platelets. Both EDP and the VGVAPG peptide strongly reduced thrombus formation *in vitro* and *in vivo* in wild-type mice. Moreover, EDP and VGVAPG also prolonged tail bleeding times. The same study also reported that the regulatory role of EDP relies on a dual mechanism that involves effects on platelets, that express a functional ERC able to trigger an increase of platelet sialidase activity, and on the ability of EDP to disrupt plasma von Willebrand factor interaction with collagen. Therefore, it is tempting to speculate that EDP may rather have beneficial effects on cancer-associated thrombosis by reducing platelet aggregation and thrombus formation.

However, if EDP modulate the formation of procoagulant microparticles by malignant cells and tissue factor expression of, the major molecular driver of cancer-associated coagulopathy and thromboembolic disorders (Mitrugno et al., 2015), remains unknown so far. Additional experiments are required to better understand the overall effects of elastin degradation products on cancer-associated thrombosis.

## Conclusion

It is now admitted that ECM can directly influence cell fate and is involved in the phenotypic modulation of cells during cancer progression. Matrix-derived peptides, originating from tumor microenvironment degradation, are crucial actors involved in the pathology and a potential source of innovative therapy. Thus, among all the matrikines described up to now, bibliographic data show that elastokines and their singular receptor, present important pro-tumoral activities. Consequently, the targeting of the ERC is of particular interest as it is not only directly involved in cancer development where an important elastolysis is observed, but also in cancer-associated processes such as diabetes and thrombosis.

## Author Contributions

AS, LO, SS, AG, SB, CK, PM, HEB, BR-C, LM, LDe, LDu contributed to the writing of the paper and to its relecture. LO contributed to **Table 1**. AG contributed to **Figure 1**.

## Conflict of Interest Statement

The authors declare that the research was conducted in the absence of any commercial or financial relationships that could be construed as a potential conflict of interest.
